# Fractionated stereotactic radiotherapy for skull base tumors: analysis of treatment accuracy using a stereotactic mask fixation system

**DOI:** 10.1186/1748-717X-5-1

**Published:** 2010-01-13

**Authors:** Giuseppe Minniti, Maurizio Valeriani, Enrico Clarke, Marco D'Arienzo, Michelangelo Ciotti, Roberto Montagnoli, Francesca Saporetti, Riccardo Maurizi Enrici

**Affiliations:** 1 Department of Radiation Oncology, Sant' Andrea Hospital, University "La Sapienza", via di Grottarossa 1035-1039, 00189, Rome, Italy; 2 Department of Neuroscience, NEUROMED Institute, via Atinense 18, 86077, Pozzilli (IS), Italy; 3 Department of Physics, Sant' Andrea Hospital, University "La Sapienza", via di Grottarossa 1035-1039, 00189, Rome, Italy

## Abstract

**Background:**

To assess the accuracy of fractionated stereotactic radiotherapy (FSRT) using a stereotactic mask fixation system.

**Patients and Methods:**

Sixteen patients treated with FSRT were involved in the study. A commercial stereotactic mask fixation system (BrainLAB AG) was used for patient immobilization. Serial CT scans obtained before and during FSRT were used to assess the accuracy of patient immobilization by comparing the isocenter position. Daily portal imaging were acquired to establish day to day patient position variation. Displacement errors along the different directions were calculated as combination of systematic and random errors.

**Results:**

The mean isocenter displacements based on localization and verification CT imaging were 0.1 mm (SD 0.3 mm) in the lateral direction, 0.1 mm (SD 0.4 mm) in the anteroposterior, and 0.3 mm (SD 0.4 mm) in craniocaudal direction. The mean 3D displacement was 0.5 mm (SD 0.4 mm), being maximum 1.4 mm. No significant differences were found during the treatment (P = 0.4). The overall isocenter displacement as calculated by 456 anterior and lateral portal images were 0.3 mm (SD 0.9 mm) in the mediolateral direction, -0.2 mm (SD 1 mm) in the anteroposterior direction, and 0.2 mm (SD 1.1 mm) in the craniocaudal direction. The largest displacement of 2.7 mm was seen in the cranio-caudal direction, with 95% of displacements < 2 mm in any direction.

**Conclusions:**

The results indicate that the setup error of the presented mask system evaluated by CT verification scans and portal imaging are minimal. Reproducibility of the isocenter position is in the best range of positioning reproducibility reported for other stereotactic systems.

## Introduction

Stereotactic radiation techniques in form of radiosurgery (SRS) or fractionated stereotactic radiotherapy (FSRT) are frequently employed in patients with skull base tumors in order to increase the precision of radiotherapy and decrease the potential long-term toxicity of treatment [1-3].

FSRT using a commercially available stereotactic mask fixation system (BrainLAB AG) has been routinely used at University Hospital Sant'Andrea in patients with skull base tumors since 2006. Differing from SRS, where patients are usually immobilized by an invasive stereotactic frame and radiation is given in a one large dose, patients undergoing FSRT are immobilized in a high precision relocatable noninvasive frame, so that it is possible to administrate stereotactic irradiation in a number of small doses/fractions. So far, FSRT combines the precision of stereotactic technique with the biological advantages of conventional radiotherapy.

Different frameless stereotactic systems, including infrared camera guidance [4], dental [5-11], implanted fiducial markers [12,13], and mask fixation system [14-20] have been developed in the last two decades. An essential prerequisite of a frameless system is that patient fixation and positioning are performed with a high degree of accuracy in order to delivery a safe therapeutic radiation dose. Accuracy of patient positioning reproducibility with a stereotactic mask fixation system using both CT and portal images as in current use in our department is reported and discussed.

## Methods and materials

The commercially available frameless BrainLAB stereotactic system in conjunction with the BrainScan 5.1 planning system has been used for stereotactic irradiation. Sixteen patients treated with FSRT were involved in the study. The cases included 8 meningiomas, 6 pituitary adenomas and 2 craniopharyngiomas. All patients gave their consent to the study.

The target volume was identified on the basis of the fused CT and magnetic resonance (MR) images. The gross tumor volume (GTV) was delineated as a contrast-enhancing tumor demonstrated on MRI scans. CTV was considered the same as GTV. The planning target volume (PTV) was generated by the geometric expansion of CTV plus 4 mm. Treatment volumes were achieved with 5-8 noncoplanar beams shaped using a micromultileaf collimator (MLC). All patients were treated on a 6-MV LINAC with a 120 leaf MLC (Varian Clinac 600 DBX). Treatment dose varied between 45 and 55 Gy in 25-33 fractions over 5-6 and 1/2 weeks.

### FSRT procedure

**Figure 1:**
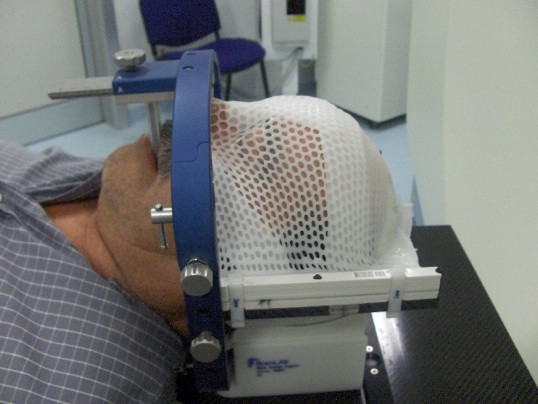
**Patient with mask fixation**. The system consists of a semicircular metal frame, an upper and a lower mask conformed to the anterior and posterior surfaces of head, two lateral carbon bars for fixing the thermoplastic mask, and a mouth bite.

The general procedure for FSRT consisted of different phases: - mask fixation; - CT localization; - treatment planning, - and CT verification. The commercial BrainLab mask fixation system (Figure 1) consisted of - a semicircular metal frame; - an upper and a lower mask conformed to the anterior (fronto-zygomatic area) and posterior surfaces (occipital and neck curvature) of head; - two lateral carbon bars for fixing the thermoplastic mask; - a mouth bite which is applied to the patient's upper dentition to avoid any head tilt movement, - and a plastic head rest. An extra rigid strip of plastic is applied across the nose-bridge, underneath the upper mask, to avoid any head rotation. Following fabrication the patient remained in the mask for 30 minutes to minimize the potential thermoplastic shrinkage during cool.

During the CT localization, a localizer box was mounted to the BrainLAB mask system in order to provide a three-dimensional (3D) stereotactic coordinate array for target localization. The patient is laid on the CT couch with the system secured onto a custom-made platform. CT imaging was performed using the GE 16-slice scanner. CT (General Electric Medical System) scaning was done in spiral mode using a pitch of 0.75, and slices in thickness and spacing of 1.2 mm acquired throughout the entire cranium. Tube voltage and tube potential were set at 130 kV and 300 mA to obtain high quality 1.2 mm reconstructed slices.

CT localization set was imported into the planning system (BrainScan) and stereotactic localization was performed by the software by identifying the location of six localizer rods on the outside surfaces of the right, left, and anterior walls of the localizer box. Localization establishes the 3D stereotactic coordinate system for treatment planning and delivery. After volume contouring, treatment planning and optimization, the patient began the treatment. During treatment, a target positioner box permitted to align patients to the treatment position. The target positioner box consisted of a skeletal aluminium box attached onto the mask system. The position of the treatment isocenter and the shapes of the beam projections were generated on four pieces of transparency by the planning system, and were attached to the anterior, superior and lateral sides of the target positioner box to mark the isocenter. The patients were then positioned in the treatment room by aligning the isocenter of the target positioner box with the room lasers.

The accuracy of patient's head immobilization with the stereotactic mask was assessed by serial CT scans by comparing the isocenter position between CT localization and CT verification. CT verification scans in the frame were taken immediately before and every 2 weeks during the treatment using slices in thickness and spacing of 1.2 mm acquired throughout the entire cranium.

CT localization and CT verification were fused employing a fusion algorithm included in the BrainLAB planning system and the isocenter shift calculated [21]. Firstly, the verification CT set is imported in the planning system and localized automatically by the planning software through identification of the stereotactic fiducials. Since this step defines the stereotactic coordinates of all brain structures with the respect to the localizer box, errors in patient repositioning will cause a mismatch of isocenter. In the second step the localization CT (planning CT) and verification CT scans were fused, and the anatomy co-registered using the CT verification as reference CT. Finally, the new coordinates of the isocenter were recorded, and isocenter shift between verification and planning CT calculated. Deviations of isocenter coordinates in each direction were measured as mean ± standard deviation (SD) for all patients. The 3D displacement determined by the square root of the sum of squares of the displacements seen in the 3 directions was calculated. The amount of isocenter shift of serial CT scans was assessed using analysis of variance (ANOVA) for repeated measures.

**Figure 2:**
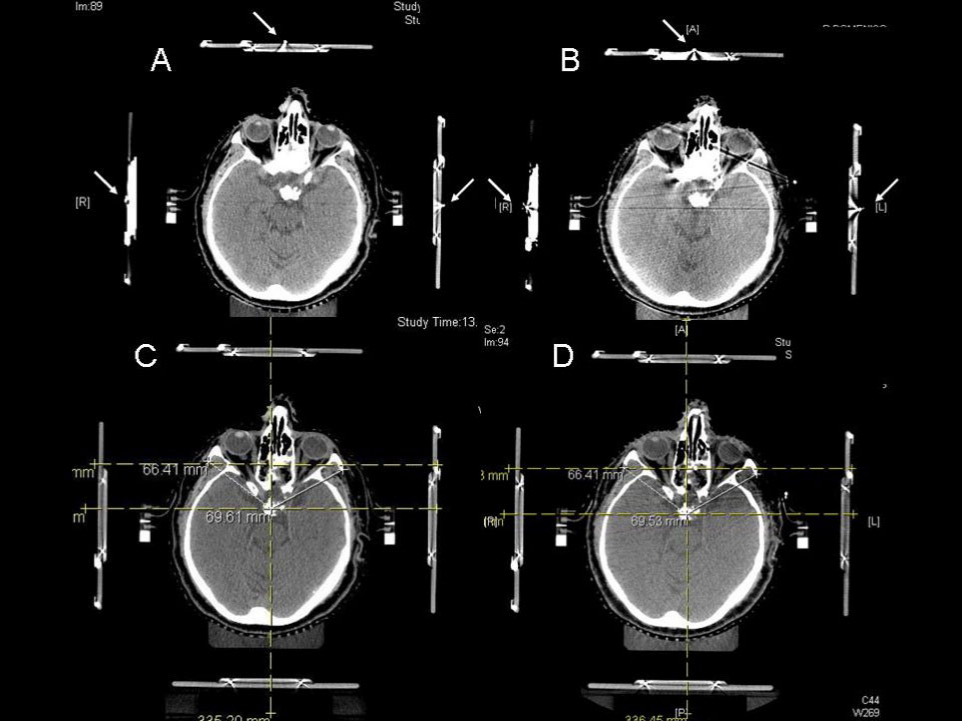
**Verification of isocenter position accuracy**. During CT localization (**A**) and CT verification (**B**) the patient is positioned on the CT couch with the target positioner box aligned with anterior and lateral lasers using the radio-opaque markers (arrows). The amount of isocenter shift between CT localization (planning CT) (C) and CT verification (D) in relation to anatomical skull base cranial structures was then evaluated directly on the CT scans.

During CT localization and CT verification 3 radiopaque markers were positioned outside the surface of the localizer box and aligned with both anterior and lateral lasers in order to reproduce the patient position. This alignment permits to assess the repositioning accuracy of BrainLAB mask by evaluating the shift of isocenter position between CT localization (planning CT) and CT verification in relation to anatomical skull base cranial structures directly on CT slices using the GE 16-slice scanner CT console, and this procedure is currently used in clinical practice before stereotactic treatments (Figure 2).

### Treatment set-up and verification by portal imaging

Before treatment, anteroposterior and right lateral radiographs were generated in the simulator room and exported to the Portal Vision^® ^(Varian Medical Systems, Palo Alto, Ca, USA) to be used as reference images. Five-six points for anatomy matching were drawn on the reference images, including superior orbital ridge and roof, pituitary fossa, frontal and occipital bones. Patients in both simulator and treatment room were positioned by aligning the isocenter of the target positioner box with the room lasers.

Daily portal images 9.6 × 9.6 cm acquired at 0 and 90° through the isocenter were obtained for each patient during the treatment for a total of 456 portal pair images. Portal and reference images were aligned by automatic matching (Varian portal Vision. 6.0). In the first step the field edge on portal image is automatically matched with the field aperture of the reference images, regardless of the anatomy and the points for matching. Then, the system aligns the portal and the reference images anatomically according to the defined points on the match anatomy layers. The patient misalignment visible as the difference between detected and planned field edges was automatically calculated. Matching was also reviewed and manually adjusted as appropriate by an experienced radiotherapist to give the best possible alignment using the visible anatomy.

Displacements along mediolateral, anterioposterior, craniocaudal direction, and 3D displacement were calculated. Displacement errors along the different directions were investigated as overall, systematic, and random errors according to previous reports [22], and as also recognized by the ICRU-62 report [23]. The systematic error, which describes the persistent positioning variation for an individual patient, was assessed by the SD of the mean value of the displacement along a given axis. Random errors, which are represented by day-to-day variation of displacements for individual patient, were assessed by subtraction of the systematic displacement from the observed displacement. For the whole population, the distribution of the random component displacements was determined by calculating the SD of all individual random values. Overall displacement for each direction is a combination of both systematic and random errors, and was determined by the square root of the sum of squares of the SD of systematic and random error.

Quality control procedures at the CT scanner, simulation room and linear accelerator were performed. The accuracy of coincidence of the radiation isocenter of the treatment unit and the laser-defined room coordinate system for patient alignment (TC scanner, simulator and treatment rooms) resulted within 1 mm.

## Results

### CT verification

Sixteen patients were evaluated in the study for a total of 64 verification CT scans. The relocation accuracy of the isocenter determined from the CT verification before the treatment is shown in Table 1. The mean measured isocenter displacements were 0.1 mm (SD 0.3 mm) in the lateral direction, 0.1 mm (SD 0.4 mm) in the anteroposterior, and 0.3 mm (SD 0.4 mm) in craniocaudal direction. The maximum displacement of 1.0 mm was seen in craniocaudal direction. The mean 3D displacement was 0.5 mm (SD 0.4 mm), being maximum 1.4 mm. Translational isocenter movements calculated during the treatment showed no significant differences in patient reproducibility (P = 0.4) (Table 1). Overall, patient reproducibility during the treatment showed maximum displacement of 1.2 mm in any direction, and 3D displacement < 1.5 mm.

**Table 1:** Positioning deviations of isocenter relocation at CT verification before and during radiation treatment.

	pre-treatment	2 weeks	4 weeks	end of treatment
Direction (mm)	mean (SD)	mean (SD)	mean (SD)	mean (SD)
Craniocaudal	0.3 (0.4)	0.3 (0.5)	0.3 (0.6)	0.4 (0.5)
Mediolateral	0.1 (0.3)	0.1 (0.4)	0.2 (0.4)	0.2 (0.4)
Anteroposterior	0.1 (0.4)	0.2 (0.4)	0.2 (0.4)	0.2 (0.5)
3D-displacement	0.5 (0.4)	0.5 (0.4)	0.6 (0.4)	0.6 (0.5)

SD, standard deviation

### Portal imaging

The mean and SD of treatment setup errors for all patients as measured from portal imaging are summarized in Table 2. The systematic, random and overall SD components of isocenter displacements along the medioateral, anterior-posterior and cranial-caudal directions are reported along with the 3D displacement. The overall displacements of the isocenter were 0.3 mm (SD 0.9 mm) in the mediolateral direction, -0.2 mm (SD 1 mm) in the anteroposterior direction, and 0.2 mm (SD 1.1 mm) in the craniocaudal direction. The largest overall displacement of 2.7 mm was seen in the craniocaudal direction, with 95% of displacements < 2 mm in any direction. Mean and SD of rotation errors in coronal and sagittal planes were 0.02° (SD 0.6°) and 0.03° (SD 0.5°), respectively. The mean 3D displacement was 1.5 mm with a mean SD of 0.5 mm (ranging from 0.2 to 2.8 mm).

**Table 2:** Mean and standard deviation of overall, systematic, and random setup errors at portal images during the treatment (n = 456)

		Distribution of displacements (1 SD, mm)		
		
	Overall displacement	Overall	Systematic	Random
	(mm)	(n = 456)	(n = 16)	(n = 456)
Mediolateral	0.3	0.9	0.7	0.5
Anteroposterior	-0.2	1	0.8	0.6
Craniocaudal	0.2	1.1	0.9	0.7

SD, standard deviation

## Discussion

Accuracy and reproducibility of patient repositioning is mandatory for FSRT. Several non-invasive stereotactic fixation systems have been developed based on masks [14-20], bite blocks [5-11] or infrared camera guidance [4]. We used a commercial stereotactic system based on a thermoplastic mask, assessing the accuracy of isocenter relocation by serial CT scans and portal imaging. The accuracy of isocenter relocation evaluated by fusion between localization and verification CT scans was less than 1.5 mm, with the largest displacement of 1.2 seen in the cranial-caudal direction. Notably, the accuracy was maintained over the 6 weeks treatment, suggesting that is appropriate to consider an isocenter shift within 2 mm during the planning process. In our study the calculation of isocenter displacement was based on fused CT images, however the accuracy of patient immobilization was also evaluated by manually superimposing the verified CT onto the reference CT using the brain structures [18]. We obtained a maximum displacement of 1.5 mm in any direction (data not shown), and currently this procedure is routinely employed in our department before stereotactic treatments.

Although results from different studies are difficult to compare because of different measuring and statistical methods applied, our positioning data are in the same range or better than other non-invasive fixation systems [5-20]. A number of studies reported on the accuracy of similar mask fixation systems evaluated by CT verification [5,6,10,11,14-16,18,19]. Using the BrainLAB stereotactic mask fixation system Wong et al. [18] reported a mean and maximum 3D displacements at the isocenter of 0.7 and 2.5 mm, respectively. Willner et al [15] reported a mean 3D displacement of 2.4 mm and SD of 1.3 mm, and similar results have been shown by others [6,14,16,19]. Using a bite block immobilization Kumar et al [11] reported an accuracy of isocenter relocation at CT verification of 0.7 mm, with a range between 0.1-1.4 mm, being similar to previous reported studies from the Royal Marsden [5,6,9]. So far, as for other radiotherapy units, CT verification represents an essential part of our FSRT quality assurance and is routinely used in our institution.

Since patient relocation evaluated by comparison of localization and verification CT scans does not include errors which are related to the treatment unit as laser alignment, machine and couch accuracy, we have evaluated setup accuracy by daily portal images. Orthogonal simulator images through isocenter were used as reference images because the advantage of sharp contrast. Mean and SD of displacements for each direction, were 0.3 mm (SD 1 mm) in the mediolateral direction, -0.2 mm (SD 1 nm) in the anteroposterior direction, and 0.3 mm (SD 1.2 mm) in the craniocaudal direction. The mean 3D displacement was 0.44 mm (SD 1.9 mm), ranging from 0.2 to 2.8 mm. Rotational movements deviations on coronal and sagittal planes showed only minimal rotational errors. A similar maximum mean rotation in the repositioning accuracy of FSRT in any direction less than 0.6 degrees has been reported by several authors using either mask or bite fixation system [10,11,17,18]. Minor rotational deviations for tumors in central parts of the head as in our study are associated with smaller isocenter shift than for tumors located in posterior fossa or lateral parts of brain, and significant changes of isocenter position are unlikely [24].

In the ICRU-62 report the overall standard deviation for PTV margin calculation is determined by quadratically adding SD for systematic errors in the patient group (Σ) and SD of distribution of the random errors (σ), although different models to calculate geometric uncertainties have been proposed [25,26]. Applying the formula CTV-to-PTV margin = 2 Σ + 0.7 σ as proposed by Stroom et al [25] we obtained a maximum value of 2.3 mm. The criterion to derive this recipe was that on average more than 99% of the CTV should at least get 95% of the dose. Van Herk et al. [26] defined a similar margin recipe for the CTV to PTV expansion (2.5 Σ + 0.7 σ) based on absorbed dose to CTV, equivalent uniform dose and tumor control probability. Applying this formula we found a value of 2.8 mm.

According to the reported results, currently in our clinical practice we use a margin from GTV to PTV expansion of 3 mm, following the above protocol. If no setup error greater than 2 mm in any one direction by portal imaging is observed during the first week, imaging will take place thrice a week for the remainder of the course. If an error more than 2 mm occurs, portal images are acquired on daily basis. In case of persistent errors patient is re-planned and margin between GTV and PTV increased from 3 to 4 mm. Larger systematic errors more than 3 mm would necessitate a repeat of the entire planning process.

## Conclusion

In conclusion, the reproducibility of the isocenter using the present mask fixation system is in the best range of positioning accuracy reported for other non-invasive fixation system for fractionated stereotactic irradiation. CT verification scans to estimate setup reproducibility result in high accuracy of isocenter relocation and is essential part of our FSRT quality assurance. Portal imaging which include couch, laser alignment and machine errors confirms the accuracy of mask fixation system showing a mean 3D setup error of 1.5 mm with a SD of 0.5 mm. A margin from GTV to PTV expansion of 3 mm seems appropriate to compensate for all possible set-up errors and is currently used in our clinical practice.

## Competing interests

The authors declare that they have no competing interests.

## Authors' contributions

GM conceived of the study, participated in its design and coordination, and drafted the manuscript. MV and EC participated in study design, analysis and interpretation of data, and helped to draft the manuscript.

MDA and MC performed the statistical analysis and carried out all CT evaluations. RM and FS participated in acquisition and analysis of data. RME critically reviewed/revised the article. All authors read and approved the final manuscript.
